# Functional Coupling of Slack Channels and P2X3 Receptors Contributes to Neuropathic Pain Processing

**DOI:** 10.3390/ijms22010405

**Published:** 2021-01-02

**Authors:** Ruirui Lu, Katharina Metzner, Fangyuan Zhou, Cathrin Flauaus, Annika Balzulat, Patrick Engel, Jonas Petersen, Rebekka Ehinger, Anne Bausch, Peter Ruth, Robert Lukowski, Achim Schmidtko

**Affiliations:** 1 Institut für Pharmakologie und Klinische Pharmazie, Goethe-Universität Frankfurt am Main, 60438 Frankfurt am Main, Hessen, Germany; Metzner@em.uni-frankfurt.de (K.M.); Zhou@stud.uni-frankfurt.de (F.Z.); flauaus@em.uni-frankfurt.de (C.F.); Balzulat@em.uni-frankfurt.de (A.B.); p.engel@em.uni-frankfurt.de (P.E.); J.Petersen@em.uni-frankfurt.de (J.P.); schmidtko@em.uni-frankfurt.de (A.S.); 2 Pharmakologie, Toxikologie und Klinische Pharmazie, Institut für Pharmazie, Universität Tübingen, 72076 Tübingen, Baden-Württemberg, Germany; rebekka.dieter@uni-tuebingen.de (R.E.); anne.bausch@rpt.bwl.de (A.B.); peter.ruth@uni-tuebingen.de (P.R.); robert.lukowski@uni-tuebingen.de (R.L.)

**Keywords:** Slack, P2X3, dorsal root ganglia, neuropathic pain, mice

## Abstract

The sodium-activated potassium channel Slack (K_Na_1.1, Slo2.2, or Kcnt1) is highly expressed in populations of sensory neurons, where it mediates the sodium-activated potassium current (I_KNa_) and modulates neuronal activity. Previous studies suggest that Slack is involved in the processing of neuropathic pain. However, mechanisms underlying the regulation of Slack activity in this context are poorly understood. Using whole-cell patch-clamp recordings we found that Slack-mediated I_KNa_ in sensory neurons of mice is reduced after peripheral nerve injury, thereby contributing to neuropathic pain hypersensitivity. Interestingly, Slack is closely associated with ATP-sensitive P2X3 receptors in a population of sensory neurons. In vitro experiments revealed that Slack-mediated I_KNa_ may be bidirectionally modulated in response to P2X3 activation. Moreover, mice lacking Slack show altered nocifensive responses to P2X3 stimulation. Our study identifies P2X3/Slack signaling as a mechanism contributing to hypersensitivity after peripheral nerve injury and proposes a potential novel strategy for treatment of neuropathic pain.

## 1. Introduction

Neuropathic pain resulting from a lesion or disease of the nervous system affects 6.9–10% of the general population [1]. It is poorly managed by existing analgesic drugs, with less than half of patients achieving satisfactory pain relief [2]. Therefore, determining the molecular constituents of neuropathic pain processing remains an important endeavor of pain research [3,4,5].

The transmission and processing of pain rely critically on the activities of ion channels that are expressed in sensory neurons. Over the past few years, several ion channel subtypes have been implicated in pain signaling and are being pursued as novel targets for analgesic therapy [6]. Among the potential targets for treatment of neuropathic pain, the sodium (Na^+^)-dependent potassium (K^+^) channel Slack (also termed K_Na_1.1, Slo2.2, or Kcnt1) has gained recent interest. Slack is only weakly voltage-dependent and activated by intracellular Na^+^ [7,8,9,10], whereas its activity is inhibited by divalent cations that modify channel gating by an allosteric mechanism [11]. Slack is highly expressed in the isolectin B4 (IB4)-binding, non-peptidergic subpopulation of C fiber sensory neurons, and accumulating evidence implicates Slack in the processing of pain [12,13,14,15,16,17,18,19].

In a previous study, we generated Slack mutant mice and observed increased pain hypersensitivity of global and sensory neuron-specific Slack knockouts in models of neuropathic pain [15]. By contrast, their behavior in models of inflammatory and acute nociceptive pain was normal [15,18], suggesting a specific function of Slack in neuropathic pain processing. However, the mechanisms that regulate Slack activity in neuropathic pain remain elusive.

Here, we further characterized the functions of Slack in pain processing. We report that the potassium currents generated by Slack in non-peptidergic C fiber sensory neurons are diminished after peripheral nerve injury. Furthermore, we provide evidence that Slack is closely associated with ATP-sensitive P2X3 receptors, which are highly expressed in non-peptidergic C fiber sensory neurons and play an important role in peripheral sensitization during chronic pain (for review, see [20,21]. Our data suggest that a P2X3-mediated ion influx modulates the activity of Slack and thus regulates neuropathic pain sensitivity.

## 2. Results

### 2.1. Slack^-/-^ Mice Show Increased Neuropathic Pain Behavior after Peripheral Nerve Injury

**Figure 1:**
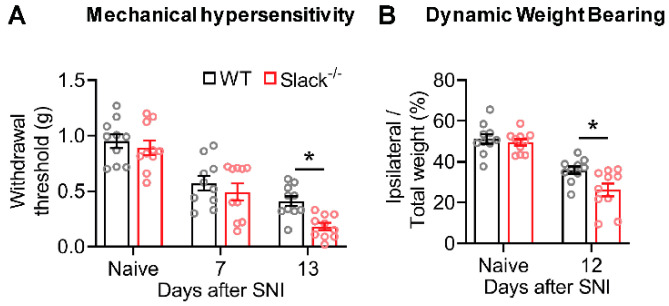
Neuropathic pain behavior is increased in Slack^-/-^ mice. (**A**) paw withdrawal latencies of Slack^-/-^ and wild-type (WT) mice after mechanical stimulation with von Frey filaments (up-and-down method) in the spared nerve injury (SNI) model of neuropathic pain (*n* = 10 animals per group). Thirteen days after SNI, Slack^-/-^ mice showed increased mechanical hypersensitivity compared to WT littermates (two-way ANOVA; *p* = 0.0312; WT versus Slack^-/-^). (**B**) percentage of weight bearing on the ipsilateral hind paw relative to both hind paws in Slack^-/-^ and WT mice (*n* = 10 animals per group), as assessed using a dynamic weight-bearing device. Twelve days after SNI, the weight-bearing reduction was more pronounced in Slack^-/-^ mice compared to WT littermates (two-way ANOVA; *p* = 0.0464; WT versus Slack^-/-^) Bars denote mean ± SEM. * *p* ˂ 0.05.

To evaluate the role of Slack in neuropathic pain processing, we exposed Slack^-/-^ and wide-type (WT) mice to the spared nerve injury (SNI) model of experimental neuropathy, wherein the tibial and common peroneal nerves are ligated and transected while the sural nerve is left intact. Thirteen days after SNI, both genotypes developed mechanical hypersensitivity in the lateral hind paw region (innervated by the spared sural nerve), as indicated by decreases in mechanical thresholds in the von Frey test (Figure 1A). Notably, the extent of SNI-induced mechanical hypersensitivity was markedly increased in Slack^-/-^ mice as compared to WT littermates (Figure 1A). These data confirm our findings in an earlier study, in which the mechanical hypersensitivity was measured using another method (Dynamic Plantar Aesthesiometer [15]). In addition, we assessed non-reflexive pain behavior using a dynamic weight-bearing device. As shown in Figure 1B, weight bearing on the ipsilateral hind paw was reduced after SNI in both genotypes. However, the weight-bearing reduction was more pronounced in Slack^-/-^ mice as compared to WT littermates, indicating an increased neuropathic pain behavior in the absence of Slack. Furthermore, control experiments in dorsal root ganglia (DRGs) of naive WT and Slack^-/-^ mice revealed similar mRNA expression levels of related potassium channels (Supplementary Figure S1A) and a similar percentage of DRG neuron populations positive for standard markers (Supplementary Figure S1B) in both genotypes, suggesting that there were no general impairments that might account for the observed behavioral phenotype in Slack^-/-^ mice. Together, these data support an inhibitory role for Slack in the modulation of neuropathic pain.

### 2.2. Slack-Mediated Potassium Currents in Sensory Neurons are Reduced after Nerve Injury

**Figure 2:**
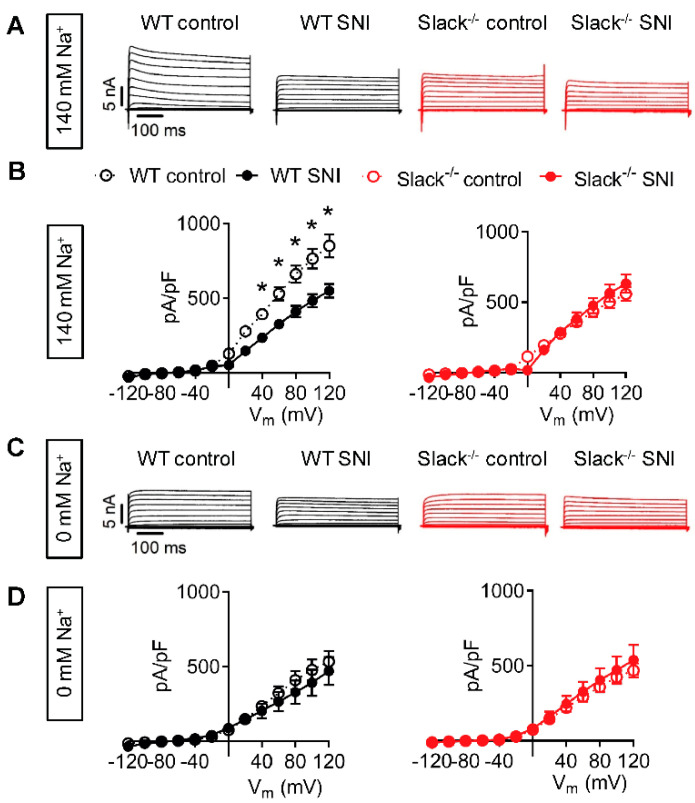
Slack-mediated potassium currents in sensory neurons are reduced after SNI. (**A**,**B**) Representative outward K^+^ current (I_K_) traces (**A**) and associated current-voltage (I-V) curves (**B**) from whole-cell voltage recordings on IB4-positive lumbar (L4-L5) dorsal root ganglion (DRG) neurons of WT (black) and Slack^-/-^ mice (red) 14–19 days after spared nerve injury (SNI). Contralateral DRG neurons were used as control. Recordings shown in (**A**) and (**B**) were performed in the presence of 140 mM NaCl in the external solution, i.e., under physiological conditions. *n* = 21–29 cells per group. Repeated ANOVA measures followed by Fisher’s Least Significant Difference test; WT control versus WT SNI: *p* = 0.0062; Slack^-/-^ control versus Slack^-/-^ SNI: *p* = 0.4374. (**C**,**D**) Representative I_K_ traces (**C**) and associated I-V curves (**D**) in the same experimental setting as shown in (**A**) and (**B**), however, after replacement of NaCl by 140 mM choline chloride in the external solution to obtain Na^+^-free conditions. *n* = 7–9 cells per group. Repeated ANOVA measures: WT control versus WT SNI: *p* = 0.1825; Slack^-/-^ control versus Slack^-/-^ SNI: *p* = 0.6125. The data show that Na^+^-activated I_K_ (I_KNa_) in sensory neurons is carried by Slack channels and reduced after SNI. Data in (**B**) and (**D**) are mean ± SEM. * *p* ˂ 0.05.

In an earlier study we demonstrated that Slack is highly expressed in DRG neurons and that the vast majority (86.5%) of Slack-positive cells bind IB4, whereas 12.6% are positive for calcitonin gene-related peptide (CGRP), two markers of non-peptidergic and peptidergic C fiber sensory neurons, respectively [15]. Moreover, Slack has been shown to generate a sodium-activated outward potassium current (I_KNa_) in whole-cell voltage-clamp recordings on IB4-positive sensory neurons of naive mice [15,18,22]. To examine whether Slack-mediated I_KNa_ is altered after peripheral nerve injury, we performed whole-cell patch-clamp recordings on IB4-positive sensory neurons of WT and Slack^-/-^ mice 14–19 days after SNI. At a holding potential of −70 mV, a series of 500 ms-long test pulses ranging from −120 to +120 mV in intervals of 20 mV were applied in the presence of 140 mM extracellular NaCl. In sensory neurons of WT mice, the amplitude of the total outward K^+^ current (I_K_) was significantly reduced after the nerve injury compared to the non-injured control (Figure 2A,B). By contrast, the I_K_ amplitude in sensory neurons of Slack^-/-^ mice was at the level of WT mice post-SNI and not altered by SNI (Figure 2A,B). Moreover, under Na^+^-free conditions (i.e., after replacement of NaCl with choline chloride in the external solution) the I_K_ amplitude was similar in all groups (Figure 2C,D). These data suggest that Slack-mediated I_KNa_ in IB4-positive sensory neurons is reduced after peripheral nerve injury, thereby contributing to neuropathic pain hypersensitivity.

### 2.3. Unaltered Slack Expression in DRGs and the Spinal Cord after Spared Nerve Injury

**Figure 3:**
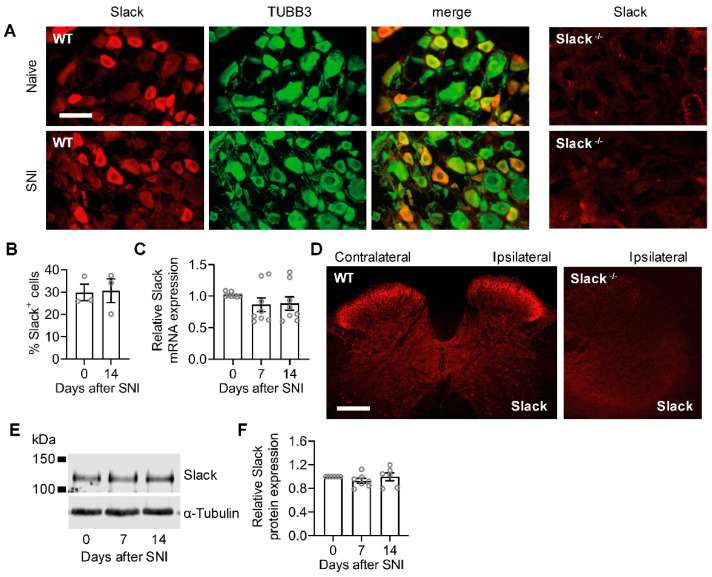
Unaltered Slack expression in WT mice after peripheral nerve injury. (**A**,**B**) Double-labeling immunostaining of Slack and the neuronal marker anti-βIII-tubulin (TUBB3) in DRGs of naive WT mice and 14 days after SNI. Colocalization of Slack and TUBB3 appears in yellow. The staining suggests that Slack is exclusively localized to neurons and that its distribution is not altered in response to the injury. The absence of Slack immunoreactivity in DRGs of Slack^-/-^ mice confirms the antibody specificity (**A**). The percentage of Slack-immunoreactive DRG neurons of all βIII-tubulin-stained neurons is similar in naive mice and 14 days after SNI ((**B**); 1833 and 1630 cells counted, respectively; *n* = 3 mice per group). Student’s t-test: *p* = 0.9130. (**C**), Quantitative RT-PCR experiments showed that Slack mRNA levels are not altered in DRGs 7 or 14 days after SNI as compared to naive control animals (*n* = 8 mice per group). One-way ANOVA: *p =* 0.1433. (**D**), Immunostaining in the lumbar spinal cord of WT mice 14 d after SNI shows similar Slack expression in the ipsilateral and contralateral dorsal horn. The absence of Slack immunoreactivity in the spinal cord of Slack^-/-^ mice confirms the antibody specificity. (**E**,**F**) A Western blot of spinal cord extracts shows similar Slack protein (140 kDa) expression in naive mice and 7 or 14 days after SNI surgery (**E**). Uncropped original image is shown in Figure S2A. Quantification is shown in (**F**). Alpha-tubulin was used as a loading control. One-way ANOVA: *p =* 0.4446. Bars denote mean ± SEM. Scale bars: 50 µm (**A**), 200 µm (**D**).

We next asked whether a downregulation of Slack expression in sensory neurons might underlay the reduced I_KNa_ after SNI in WT mice, because various potassium channels are downregulated at the transcriptional level during neuropathic pain [6]. However, immunostaining experiments using a specific anti-Slack antibody, whose immunoreactivity is absent in tissues from Slack^-/-^ mice [15] and that does not cross-react with the closely related potassium channel Slick [18], showed that the percentage of Slack-immunoreactive sensory neurons was indistinguishable in DRGs of naive mice and 14 days after SNI (Figure 3A,B). Similarly, quantitative real-time reverse transcription PCR (qRT-PCR) analyses revealed unaltered Slack mRNA expression in lumbar DRGs 7 and 14 days after SNI (Figure 3C). Moreover, Slack immunoreactivity in the ipsilateral and contralateral dorsal horn of the lumbar spinal cord, in which the central terminals of sensory neurons terminate, was similar 14 days after SNI (Figure 3D). Accordingly, Western blot analyses of spinal cord tissue extracts did not detect any alteration of Slack expression in naive and post-SNI animals (Figure 3E,F). These data suggest that the expression of Slack channels, unlike many other potassium channels, is unaltered in sensory neurons in response to peripheral nerve injury.

### 2.4. Slack Channels Colocalize with P2X3 Receptors in Sensory Neurons

**Figure 4:**
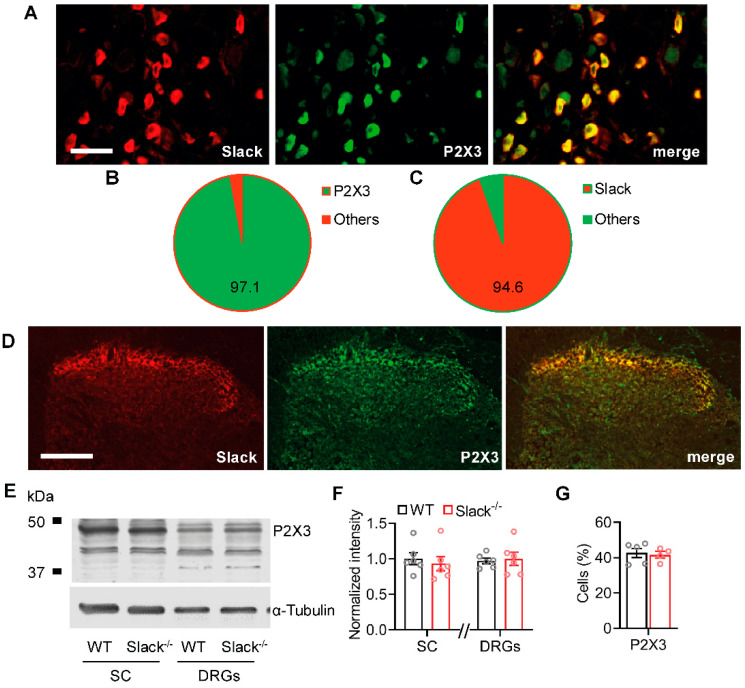
Slack channels co-localize with P2X3 receptors in sensory neurons. (**A**–**C**) Double-labeling immunostaining of Slack and P2X3 in sensory neurons (**A**) revealed that 97.1% ± 0.2% of Slack-positive DRG neurons co-stained with P2X3 ((**B**); 1203 cells counted, *n* = 3 mice) and that 94.6% ± 2.0% of P2X3-positive DRG neurons co-stained with Slack ((**C**); 1203 cells counted, *n* = 3 mice). (**D**) Double-labeling immunostaining of Slack and P2X3 in the spinal cord indicates a high degree of co-localization in the superficial dorsal horn. (**E**,**F**) Western blot of P2X3 in spinal cord (SC) and DRGs from WT and Slack^-/-^ mice demonstrates identical abundance of P2X3 in both genotypes. The uncropped original image is shown in Figure S2B. Student’s t-test: *p* = 0.5986 in the spinal cord and *p* = 0.7631 in DRGs. Alpha-tubulin was used as a loading control. (**G**) Immunostaining revealed that the percentage of DRG neurons positive for P2X3 is similar in WT and Slack^-/-^ mice. Student’s t-test: *p* = 0.4046. Bars denote mean ± SEM. Scale bars: 50 µm (**A**) and 100 µm (**D**).

The unaltered Slack expression after SNI points to inhibition of Slack channel activity underlying the reduced I_KNa_ after peripheral nerve injury. Because Slack channels are inhibited by intracellular Ca^2+^ and other divalent cations [11], we speculated that Ca^2+^-permeable ion channels in proximity to Slack channels might mediate the inhibition of I_KNa_ in sensory neurons after nerve injury. Given the enriched expression of Slack in IB4-positive sensory neurons [15,23] and its functional contribution to neuropathic pain processing, we hypothesized several reasons why Slack might interact with the ATP receptor subunit P2X3: (i) P2X3 receptors, like Slack, are almost exclusively expressed in IB4-positive sensory neurons [24], (ii) they are permeable for Ca^2+^, Na^+^, and other cations, and (iii) signaling through P2X3 receptors essentially contributes to neuropathic pain hypersensitivity [25,26,27,28]. As shown in Figure 4A–C, the co-localization of Slack and P2X3 in DRG neurons is almost complete, as 97.1% of Slack-positive DRG neurons co-stained with P2X3 and 94.6% of P2X3-positive DRG neurons co-stained with Slack. Control experiments confirmed that almost all P2X3-positive DRG neurons bind IB4 (Figure S2C), suggesting that the anti-P2X3 antibody used in our immunostaining experiment is specific. Furthermore, Slack and P2X3 are also highly co-localized in the dorsal horn of the spinal cord (Figure 4D). Moreover, Western blot control experiments demonstrated similar P2X3 protein levels in DRGs and the spinal cord of Slack^-/-^ and WT mice (Figure 4E,F) and P2X3 immunostaining detected a similar percentage of P2X3-positive DRG neurons in both genotypes (Figure 4G), suggesting that there was no compensatory regulation due to the Slack knockout that might have contributed to the observed pain behavior and neuronal potassium currents in Slack^-/-^ mice. Together, these findings point to a possible interaction of Slack and P2X3 in sensory neurons.

### 2.5. P2X3 Activation Modulates Slack-Mediated Potassium Currents In Vitro

**Figure 5:**
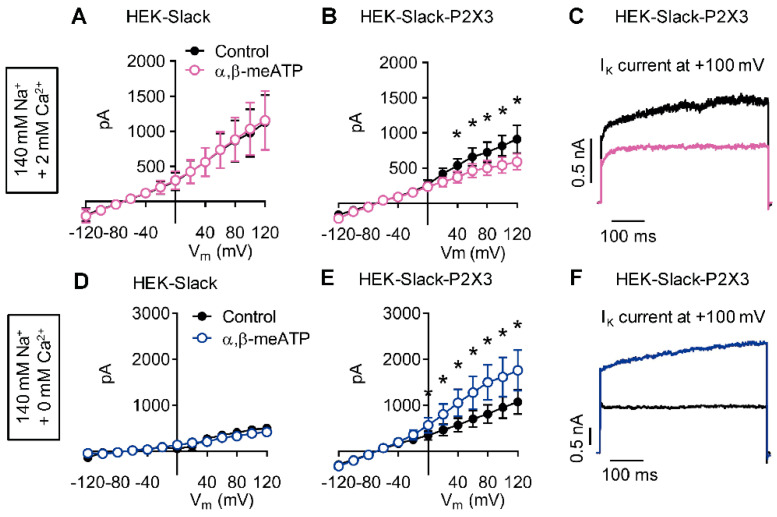
Slack-mediated potassium currents are altered by P2X3 activation in vitro. Representative I_K_ traces from whole-cell voltage-clamp recordings on HEK-Slack and HEK-Slack-P2X3 cells are shown. The current traces presented in (**A**–**C**) were recorded in the presence of 2 mM CaCl_2_ in the external solution (*n* = 9–10 cells per group), whereas those depicted in (**D**–**F**) were recorded after the replacement of CaCl_2_ by MgCl_2_ (*n* = 5–8 cells per group). Experiments were performed without (control) or with the addition of the P2X3 agonist α,β-methylene ATP (α,β-meATP; 30 µM) to the external solution. Note that the P2X3 agonist exerted opposite effects in HEK-Slack-P2X3 cells dependent on the Ca^2+^ concentration: α,β-meATP reduced I_K_ in the presence of Ca^2+^, whereas it increased I_K_ under Ca^2+^-free conditions. Data are shown as mean ± SEM. Student’s t-test, * *p* < 0.05.

We next studied the functional properties of co-expressed recombinant Slack and P2X3 receptors. For that purpose, we used a commercially available HEK-293 cell line stably expressing human Slack channels (herein referred to as HEK-Slack cells) and transiently transfected GFP-tagged human P2X3 receptors into these cells. This strategy led to expression of P2X3 in about 40% of transfected cells (as indicated by green fluorescence in microscopy; the resulting P2X3-positive cells were referred to as HEK-Slack-P2X3 cells). We performed whole-cell patch-clamp recordings at a holding potential of −70 mV by applying series of 500 ms-long test pulses ranging from −120 to +120 mV in intervals of 20 mV. A first series of experiments were conducted using a physiological external solution (i.e., in the presence of 140 mM NaCl, 5 mM KCl, 2 mM CaCl_2_, 2 mM MgCl_2_, and 10 mM HEPES). As shown in Figure 5A–C, outward potassium currents (I_K_) were detected in both HEK-Slack and HEK-Slack-P2X3 cells. Interestingly however, the addition of the P2X3 agonist α,β-meATP (30 µM [29]) to the external solution reduced I_K_ in HEK-Slack-P2X3 cells (Figure 5B,C) but not in HEK-Slack cells (Figure 5A). These results imply that P2X3 activation, which in our experimental setting may result in an influx of Na^+^, Mg^2+^, and Ca^2+^ [30,31], is associated with inhibition of Slack activity.

A recent study revealed that Slack activity is inhibited by divalent cations [11]. We reasoned that the observed reduction of I_K_ in the presence of the P2X3 agonist might be mediated mainly by Ca^2+^, because Mg^2+^ represents a much weaker Slack inhibitor than Ca^2+^ [11]. To test this hypothesis, we performed experiments under Ca^2+^-free conditions, i.e., after replacement of Ca^2+^ in the external solution by Mg^2+^. Strikingly, in the absence of Ca^2+^ α,β-meATP increased I_K_ in HEK-Slack-P2X3 cells but not in HEK-Slack cells (Figure 5D–F). We assumed that the increased I_K_ was attributed to an exaggerated, Na^+^-mediated activation of Slack channels due to the lack of inhibition by Ca^2+^. Altogether, these data suggest that Slack-mediated I_KNa_ may be bidirectionally modulated by a P2X3-driven ion influx.

### 2.6. P2X3-Mediated Ca^2+^ Influx Is Unaltered in Sensory Neurons of Slack^-/-^ Mice

**Figure 6:**
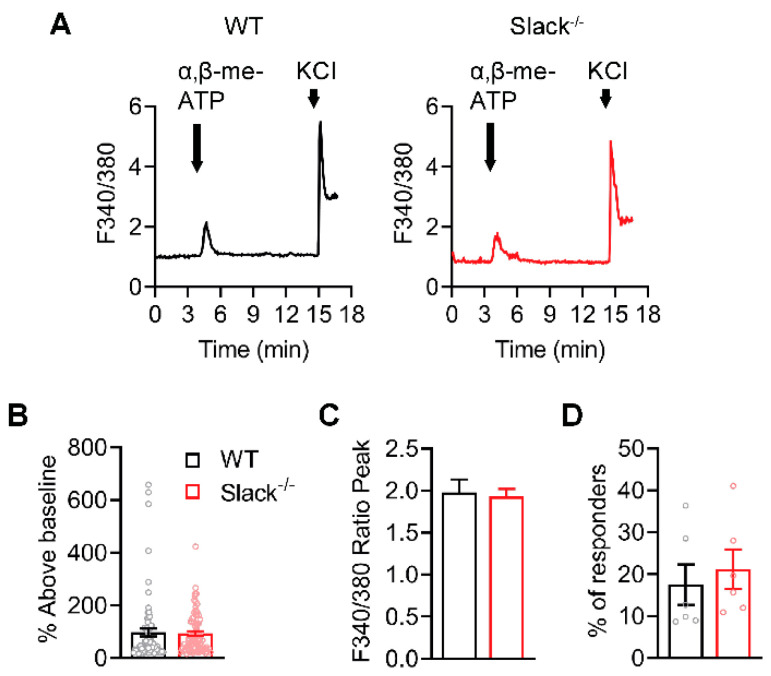
P2X3-mediated Ca^2+^ traces are normal in sensory neurons of Slack^-/-^ mice. (**A**) Representative examples of Fura-2 ratiometric Ca^2+^ traces in DRG neurons of WT and Slack^-/-^ mice evoked by α,β-meATP (30 µM, 30 s application) and KCl (75 mM, 20 s application). Responses to KCl were used to test neuron viability. Experiments were performed in lumbar (L4-L5) DRG neurons (*n* = 402–518 neurons per group). (**B**,**C**) Quantification of the magnitude of the Ca^2+^ response to α,β-meATP stimulation with percentage above baseline ((**B**) *p* = 0.7858) and ratio peak ((**C**) *p* = 0.7858). (**D**) Quantification of the percentage of responsive neurons to α,β-meATP stimulation (*p* = 0.5966). The data show that α,β-meATP-evoked Ca^2+^ responses are similar in DRG neurons from Slack^-/-^ and WT mice. Bars denote mean ± SEM and circles show data from each neuron in (**B**) and from each mouse in (**D**). Student’s t-tests were performed.

We next investigated the Slack-P2X3 interaction in sensory neurons. To exclude the possibility that a P2X3-driven Ca^2+^ influx in sensory neurons might be generally altered by Slack deficiency, we performed control experiments to analyze the α,β-meATP-evoked Ca^2+^ influx in sensory neurons of WT and Slack^-/-^ mice. Calcium imaging in cultured lumbar DRG neurons of WT and Slack^-/-^ mice revealed that incubation with α,β-meATP (30 µM [32]) evoked a similar calcium influx in both genotypes (Figure 6A). The magnitude of the Ca^2+^ response (Figure 6B,C) and the proportion of responsive DRG neurons (Figure 6D) were comparable in DRG neurons of WT and Slack^-/-^ mice. These data confirm that Slack deficiency per se does not affect the P2X3-mediated Ca^2+^ influx in sensory neurons.

### 2.7. P2X3-Mediated Pain Behavior Is Altered in Slack^-/-^ Mice

**Figure 7:**
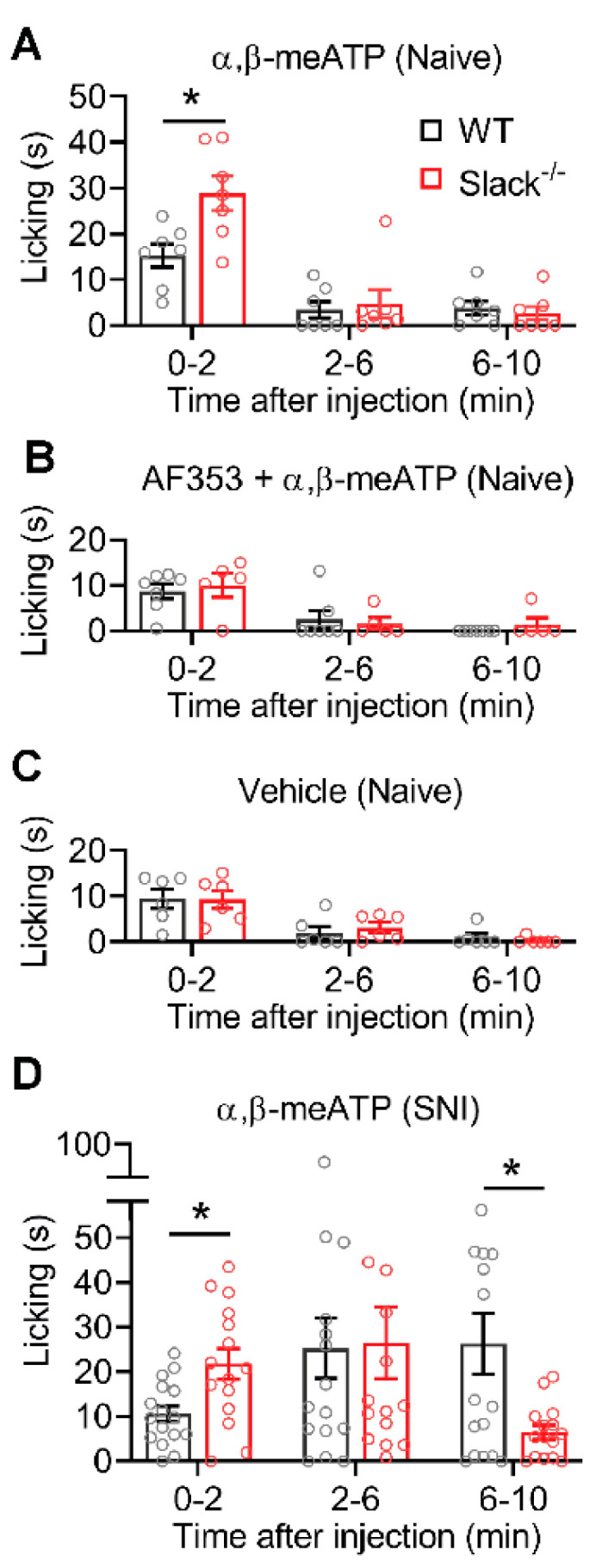
P2X3-dependent nocifensive behavior is altered in Slack^-/-^ mice. Paw-licking responses induced by paw injection of drugs are shown. (**A**) The immediate paw licking in the first 2 min after injection of α,β-meATP (12 nmol) is increased in naive Slack^-/-^ mice compared with WT mice (*n* = 7/genotype; *p* = 0.0398 in 0–2 min; *p* = 0.9800 in 2–6 min; *p* = 0.9352 in 6–10 min). (**B**) No significant differences between groups occurred when the P2X3 receptor antagonist AF353 (70 nmol intraplantar) was injected 10 min prior to α,β-meATP (*n* = 5–7/genotype; *p* = 0.9664 in 0–2 min; *p* = 0.9732 in 2–6 min; *p* = 0.7546 in 6–10 min). (**C**) Paw-licking responses after injection of the vehicle were comparable in Slack^-/-^ and WT mice (*n* = 6/genotype; *p* = 0.9999 in 0–2 min; *p* = 0.8934 in 2–6 min; *p* = 0.8709 in 6–10 min) and similar to the licking behavior after combined injection of α,β-meATP and AF353 (**B**). (**D**) When α,β-meATP (12 nmol) was injected in the ipsilateral hind paw after SNI, the paw-licking response persisted over the 10 min observation period in WT mice. In Slack^-/-^ mice, the paw licking was increased in the first 2 min and decreased from 6–10 min compared with WT mice (*n* = 16/genotype; *p* = 0.0262 in 0–2 min; *p* = 0.9993 in 2–6 min; and *p* = 0.0362 in 6–10 min). Two-way ANOVA tests were performed. Bars denote mean ± SEM. * *p* ˂ 0.05.

We then assessed a functional P2X3/Slack interaction in behavioral experiments in vivo. We injected α,β-meATP (12 nmol [26]) into a hind paw of naive WT and Slack^-/-^ mice and analyzed the resulting paw-licking response over 10 min. Of note, during the first 2 min, the paw licking induced by α,β-meATP was significantly increased in Slack^-/-^ mice as compared to WT mice (Figure 7A). Virtually no paw licking was observed at later time points (2–10 min) in both genotypes (Figure 7A), confirming earlier reports that the immediate nocifensive response to intraplantar α,β-meATP is relatively short-lived in naive animals [27]. After pretreatment with the selective P2X3 receptor antagonist AF353 (70 nmol intraplantar [33,34]) 10 min prior to the α,β-meATP injection, the paw-licking response was reduced, and no significant differences between groups were detected (Figure 7B). A similar paw-licking behavior occurred after injection of the vehicle (0.9% NaCl; Figure 7C), suggesting that the behavioral responses to the α,β-meATP injection shown in Figure 7A were mediated by P2X_3_ receptors. These data indicate that Slack channels control the immediate pronociceptive effects of P2X_3_ receptor activation in naive mice in vivo.

We next analyzed the P2X3/Slack interaction during neuropathic pain using the SNI model. For that purpose, α,β-meATP (12 nmol) was injected into the dorsolateral region of the ipsilateral hind paw 2–3 weeks after SNI. As shown in Figure 7D, in WT mice the paw-licking response after injection of α,β-meATP persisted over the 10 min observation period, which is in line with the sensitization of P2X3 receptors after traumatic injury that was reported in previous studies [26,27,35]. In Slack^-/-^ mice, the paw-licking response was increased compared to WT mice in the first 2 min (with a similar licking time as observed in naive mice, see Figure 7A). During 2–6 min after the injection, the licking response was similar in Slack^-/-^ and WT mice (and increased compared to naive mice, see Figure 7A), pointing to a sensitization of P2X3 receptors in both genotypes. Interestingly however, at late stages (6–10 min) the paw licking in Slack^-/-^ mice was lower than in WT mice (Figure 7D). These data suggest that Slack facilitates the P2X3 receptor sensitization at late stages, which is in accordance with a Ca^2+^-driven inhibition of Slack channel activity in vivo.

## 3. Discussion

**Figure 8:**
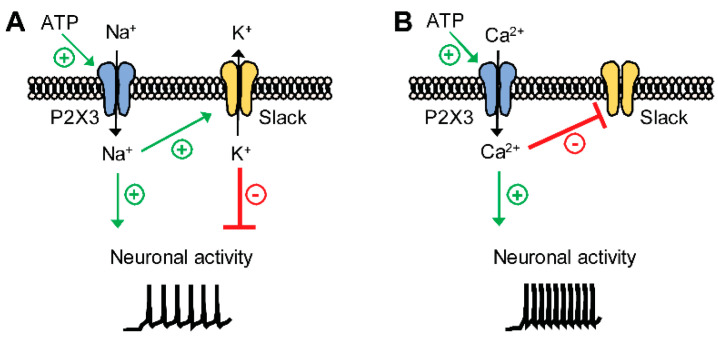
Proposed model demonstrating the functional coupling of Slack channels and P2X3 receptors in IB4-positive sensory neurons. Activation of P2X3 receptors by ATP may lead to an influx of Na^+^, Ca^2+^, or both into sensory neurons. (**A**) P2X3-mediated Na^+^ influx activates Slack, which results in K^+^ efflux and thus partial inhibition of neuronal activity. (**B**) P2X3-mediated Ca^2+^ influx inhibits Slack, which leads to increased neuronal activity due to the lack of inhibition.

Here, we provide further support that Slack plays an important role in neuropathic pain processing. We uncovered Slack/P2X3 signaling as a new mechanism contributing to neuropathic pain processing. Strikingly, our data suggest that activation of P2X3 receptors may both activate and inhibit Slack, depending on P2X3 ion permeability. The main findings from this study are highlighted in a schematic diagram in Figure 8. Collectively, these data add to the complexity of ion channel regulation in sensory neurons during persistent pain.

Slack channels are widely distributed in the central and peripheral nervous systems. Previous studies detected particularly high Slack expression levels in IB4-positive, non-peptidergic sensory neurons [14,15,23,36], and behavioral analyses of global and sensory neuron-specific Slack knockout mice uncovered an inhibitory function of Slack in the processing of neuropathic pain [15]. These observations are further supported by a recent clinical trial using the Slack activator loxapine [19], pointing to Slack as a potential novel target to alleviate chronic pain in humans. We here confirm that Slack modulates neuropathic pain processing, because Slack^-/-^ mice demonstrated an increased mechanical hypersensitivity (assessed using von Frey filaments) and a decreased weight distribution on the ipsilateral hind paw (assessed using dynamic weight bearing) after peripheral nerve injury.

Our data further expand the role of Slack in neuropathic pain by demonstrating that I_KNa_ in IB4-positive sensory neurons, which is mainly driven by Slack, is reduced after peripheral nerve injury. In general, under neuropathic pain conditions there is a reduction in the expression of several subtypes of potassium channels that gives rise to hyperexcitability [6,37]. Prominent examples of downregulated potassium channels in DRG neurons after peripheral nerve injury include Kv9.1 [3,38], Kv1.1, and Kv1.2 [39,40]. Our observation that, unlike these channels, Slack expression in DRGs and the spinal cord is not altered after peripheral nerve injury further supports the idea that Slack might be a target for pain therapy.

Our study uncovered P2X3 as a previously unrecognized signaling pathway that regulates Slack activity. Previously identified Na^+^-permeable ion channels that may activate Slack include AMPA receptors [41] and Na_v_1.8 channels [22]. It has been shown that Na^+^ entering a neuron via a small but persistent sodium current may be effective in activating I_KNa_, even in the absence of bulk internal Na^+^ [10]. Furthermore, a recent study demonstrated that Slack is inhibited by divalent cations, most likely by an allosteric mode of action involving a His residue downstream S6 that is analogous to an inhibitory cation binding site in cyclic nucleotide gated channels [11]. We here demonstrate that a P2X3-driven ion influx may both stimulate and inhibit Slack activity: (i) In HEK-Slack-P2X3 cells, the addition of the P2X3 agonist α,β-meATP to the extracellular solution reduced I_K_ in presence of Ca^2+^, whereas it increased I_K_ under Ca^2+^-free conditions. (ii) In naive mice, paw injection of α,β-meATP induced paw licking in a Slack-dependent manner that was blocked by pretreatment with the P2X3 antagonist AF353. The reduced paw licking of WT mice as compared to Slack^-/-^ mice suggests that a P2X3-driven Na^+^ influx activates Slack, which in turn limits the nocifensive behavior. (iii) After SNI, the paw licking evoked by α,β-meATP was ameliorated in Slack^-/-^ mice at late stages (6–10 min). The longer lasting paw licking in WT mice indicates that a P2X3-driven Ca^2+^ influx might inhibit Slack, thereby facilitating the nocifensive behavior. However, it should be taken into account that α,β-meATP is an agonist not only at P2X3 but also at other P2X receptors [42,43] and that AF353 also inhibits P2X2/3 heteromeric receptors [34]. Hence, P2X receptors different from P2X3 might theoretically contribute to the observed pain behavior after α,β-meATP administration.

It is important to note that, in addition to ion influx, Slack activity may be modulated by protein kinase A [13,17], NAD^+^ [12], a Na^+^ sensor in the RCK2 domain [44], and TMEM16C [14]. On the other hand, different firing modalities have been detected after incubation of trigeminal neurons with α,β-meATP, suggesting that the co-expression of P2X3 with different interacting proteins might affect the firing responses [45]. In general, several factors have been identified that may interact with P2X receptors. In particular, P2X receptors may physically interact with members of the Cys-loop receptor family such as nicotinic, GABA_A/C_, and 5-HT_3A_ receptors [46,47,48,49,50]. A recent study revealed that P2X3 receptors in sensory neurons tightly associate with acid-sensing ion channels, thereby forming a protein complex that mediates unidirectional inhibition [51]. Furthermore, protein modifications such as glycosylation can affect the function of ion channels at the level of subunit assembly, protein trafficking, ligand binding, and channel opening. It has been shown that glycosylation also affects proper folding, oligomeric association, and compartmentalization of P2X3 receptors in neuronal cells [52,53]. Hence, the P2X3–Slack interaction we report here further contributes to the complexity of pain processing.

In conclusion, our study suggests that Slack controls the excitability of IB4-positive sensory neurons under neuropathic pain conditions and that their activity is regulated by P2X3 receptors. Thus, targeting these channels might prove to be a new direction for the treatment of neuropathic pain.

## 4. Materials and Methods

### 4.1. Animals

Generation of global Slack mutants (Slack^-/-^) by a targeted ablation of Slack gene *Kcnt1* in murine embryonic stem cells in combination with a common Cre/loxP system has been described previously [15]. Experiments were performed in mice of either sex backcrossed onto a C57BL/6N background. Littermate mice were used in all behavioral and patch-clamp studies, and were investigated by an observer blinded for the genotype and treatment of the animals. In addition, C57BL/6N mice from Charles River laboratories (Sulzfeld, Germany) were used for qRT-PCR and immunoblotting analyses. Animals were housed on a 12/12 light/dark cycle with access to food and water ad libitum. All experiments adhered to the International Association for the Study of Pain (IASP) and the Animal Research: Reporting of In Vivo Experiments (ARRIVE) guidelines, the 3Rs principles, and were approved by our local Ethics Committees for Animal Research (Regierungspräsidium Darmstadt, Germany (protocol V54-19c20/15-F95/45 with approval date 3 May 2012 and protocol V54-19c20/15-FR/1013 with approval date 17 May 2018) and Landesamt für Natur, Umwelt und Verbraucherschutz NRW, Recklinghausen, Germany (protocol 84-02.04.2015.A119 with approval date 1 October 2015)).

### 4.2. Behavioral Testing

#### 4.2.1. Spared Nerve Injury Model of Neuropathic Pain

The SNI model of neuropathic pain was performed as described previously [54,55]. Under isoflurane anesthesia and carprofen analgesia, the common peroneal and tibial nerve branches of the sciatic nerve were ligated with a 6–0 silk suture and approximately 1 mm of the two nerve branches was removed distally, whereas the sural nerve was left intact.

For testing mechanical sensitivity, mice were placed in boxes on an elevated metal mesh floor and habituated for at least 30 min before behavioral testing began. Calibrated von Frey filaments ranging from 0.40 to 39.2 mN (0.02 to 4.0 g; Ugo Basile, Gemonio, Italy) were applied to the lateral part of the hind paw (sural nerve area) until they bowed for 5 s. Only obvious withdrawal responses to the applied stimulus were recorded. The 50% withdrawal thresholds were assessed using the up-down method [56,57] and calculated using an online algorithm (https://bioapps.shinyapps.io/von_frey_app/ [58]).

An automated dynamic weight-bearing (DWB) device (Bioseb, Vitrolles, France) was used to evaluate the dynamic weight distribution in mice after SNI. The system automatically records the average weight (in grams) that each limb exerts on a floor equipped with pressure transducers. A zone was considered valid when the following parameters were detected: ≥ 0.8 g on 1 captor with a minimum of 2 adjacent captors recording ≥ 1.0 g. For testing, the mouse was placed in the chamber and allowed to move freely within the apparatus for 10 min and videotaped. After 5 min of acclimatization, the dynamic weight distribution was recorded for 5 min. In all video sequences, the positions of the paws were manually validated by the observer [59,60]. The results were expressed as percent weight borne by the ipsilateral hind paw of total weight borne by both hind paws [61].

#### 4.2.2. α,β-meATP-Induced Paw Licking

The animals were placed into a plexiglass cylinder (diameter 30 cm) and habituated for at least 30 min. α,β-Methylene adenosine 5′-triphosphate (α,β-meATP; 12 nmol in 12 µL 0.9% saline [26]; Sigma-Aldrich, Darmstadt, Germany), AF353 (70 nmol in 12 µL 0.9% saline [33]; Sigma-Aldrich), or 0.9% saline (B. Braun, Melsungen, Germany) was injected into the dorsolateral hind paw and the time spent licking the injected paw was scored for 10 min. In experiments with two compounds, AF353 was injected 10 min prior to α,β-meATP.

### 4.3. Quantitative Real-Time Reverse Transcription PCR

Mice were killed by CO_2_ inhalation and their lumbar (L4-L5) DRGs were rapidly dissected, snap frozen in liquid nitrogen, and stored at −80 °C. Total RNA was extracted under RNase-free conditions using an RNA isolation Kit (RNAqueous Micro Kit; Ambion/Life Technologies, Carlsbad, USA) according to the manufacturer’s instructions, and DNase treated for 15 min to minimize genomic DNA contamination and quantified with a NanoDrop ND-1000 spectrophotometer (NanoDrop Technologies, Wilmington, USA). cDNA was synthesized from 200 ng RNA, random hexamer primers, RT-Enhancer, and the Verso enzyme of the Verso Kit (Thermo Fisher Scientific, Frankfurt, Germany). Quantitative real-time reverse transcription PCR (qRT-PCR) was performed on a 7500 Fast Real-Time PCR System (Applied Biosystems/Life Technologies) using Taqman gene expression assays for *Kcnt1* (catalog no. Mm01330653_m1), *Kcnma1* (catalog no. Mm01119505_m1), *Kcnt2* (catalog no. Mm01284549_m1), and GAPDH (catalog no. Mm99999915_g1), all purchased from Applied Biosystems. Reactions (total volume 10 µl) were performed in duplicate or triplicate by incubating at 95 °C for 10 min, followed by 40 cycles of 15 s at 95 °C and 1 min at 60 °C. Water controls were included to ensure specificity. Relative expression of target gene levels was determined using the comparative 2^-ΔΔCt^ method, with Ct indicating the cycle number at which the signal of the PCR product crossed an arbitrary threshold set within the exponential phase of the PCR. The amount of sample RNA was normalized to GAPDH.

### 4.4. Immunostaining

Mice were killed by CO_2_ and immediately perfused intracardially with 0.9% saline, followed by 1% paraformaldehyde in phosphate-buffered saline (PBS), pH 7.4. The lumbar (L4-L5) DRGs and spinal cord were dissected and cryoprotected in 20% sucrose overnight. Tissues were frozen in tissue-freezing medium (Leica, Nussloch, Germany) on dry ice, cryostat sectioned at a thickness of 14 µm, and stored at −80 °C. For immunostaining, sections were permeabilized for 5 min in 0.1% Triton X-100 in PBS, blocked for 1 h using 10% normal goat serum and 3% bovine serum albumin (BSA) in PBS, and incubated with primary antibodies diluted in 3% BSA in PBS overnight at 4 °C or for 2 h at room temperature. The following antibodies were used: mouse anti-slo2.2 (1:400, 75-051, NeuroMab; Davis, USA) [15], rabbit anti-P2X3 (1:800, AB5895, Sigma-Aldrich, Darmstadt, Germany) [18,62], rabbit anti-CGRP (1:800, PC205L, Calbiochem/Sigma-Aldrich) [15,63], mouse anti-NF200 (1:2000, N0142, Sigma-Aldrich) [64,65] and rabbit anti-tyrosin hydroxylase (1:400, AB152, Millipore/Sigma-Aldrich). Sections were then washed in PBS and stained with secondary antibodies conjugated with Alexa Fluor 488 (AF488) or Alexa Fluor 555 (1:1200, Thermo Fisher Scientific). For staining with tubulin β 3 (TUBB3), sections were incubated with AF488-conjuagted TUBB3 (1:1000, 801203, Biolegend, San Diego, USA) in PBS. For IB4 binding, sections were incubated with AF488-conjuagated IB4 (1:400, 121411, Invitrogen/Thermo Fisher Scientific) in PBS containing 1 mM CaCl_2_·2H_2_O, 1 mM MgCl_2_, 1 mM MnCl_2_, and 0.2% Triton X-100, pH 7.4). After immunostaining, slides were immersed for 5 min in 0.06% Sudan black B (in 70% ethanol) to reduce lipofuscin-like autofluorescence [66], washed in PBS, and coverslipped. In double-labeling experiments, primary antibodies were consecutively incubated. Images were taken using an Eclipse Ni-U (Nikon, Düsseldorf, Germany) microscope equipped with a monochrome CCD camera, and were pseudocolored and superimposed. Adjustment of brightness and contrast was done using Adobe Photoshop 2020 software (Adobe Systems). Controls were performed by omitting the first or the second antibodies, or both, and by incubating tissues of Slack^-/-^ mice.

For cell counting, serial DRG sections from 3 mice per genotype were processed and at least 3 sections per DRG per animal were counted. Only cells showing staining clearly above the background were included. Specificity of Slack immunoreactivity was confirmed by simultaneous staining of co-embedded tissues of WT and Slack^-/-^ mice. The percentage of Slack-positive or P2X3-positive cells was calculated as a proportion of positive cells per total number of DRG neurons.

### 4.5. Western Blot

The lumbar (L4-L5) DRGs and spinal cord were rapidly dissected, frozen in liquid nitrogen, and stored at −80 °C until use. For Slack detection, samples were homogenized in buffer containing 100 mM Tris-HCl and 1 mM MgCl_2_, pH 8.0, combined with a protease inhibitor mixture (Pierce Protease Inhibitor Mini Tablets; Thermo Scientific). After freezing for 30 min at −80 °C, samples were homogenized again. Then a sixfold volume of sucrose buffer (250 mM sucrose, 10 mM Tris-HCl, pH 7.4, combined with protease inhibitor mixture) was added followed by centrifugation at 1000 x *g* for 20 min. The supernatant was collected and centrifuged at 20,000 x *g* for 20 min, and the resulting pellets were resuspended in sucrose buffer [15]. For P2X_3_ detection, samples were homogenized in radioimmunoprecipitation assay (RIPA) buffer containing 150 mM NaCl, 50 mM Tris, 0.5% sodium deoxycholate, 0.1% SDS, and 1% Triton X-100, pH 8.0, combined with protease inhibitor mixture, and centrifuged at 12,000 x *g* for 30 min. Extracted proteins (30 µg/lane) were separated by SDS-PAGE and blotted onto a nitrocellulose membrane. After blocking nonspecific binding sites with blocking buffer (PBS with 3% low-fat milk) for 1 h, membranes were incubated with mouse anti-slo2.2 (1:500), rabbit anti-P2X_3_ (1:500), mouse anti-α-tubulin (1:800, Millipore/Sigma-Aldrich), or mouse anti-GAPDH (1:2000; Thermo Fisher Scientific) dissolved in blocking buffer containing 0.1% Tween 20 overnight at 4 °C. After incubation with secondary antibodies (IRDye^®^ 680LT goat anti-mouse IgG_1_, IRDye^®^ 680RD goat anti-rabbit IgG, IRDye^®^ 800RD goat anti-rabbit IgG, or IRDye^®^ 800RD goat anti-mouse IgG from LI-COR Bioscience, Bad Homburg, Germany) for 1 h at room temperature, proteins were detected using an Odyssey Infrared Imaging System or a C-DiGit blot scanner (LI-COR Bioscience).

### 4.6. Patch Clamp Recordings

A DRG neuron primary cell culture was prepared as described before [15]. Briefly, mice were killed by CO_2_ inhalation, and lumbar (L4-L5) DRGs were transferred to dulbecco’s modified eagle´s medium containing 50 μg/mL gentamicin (Sigma Aldrich). Following treatment with 500 U/mL collagenase IV and 2.5 U/mL dispase II for 30 min (both from Sigma Aldrich) and 0.05% Trypsin/ethylenediaminetetraacetic acid (Gibco/Thermo Fisher Scientific) for 10 min, cells were mechanically dissociated using a pipette. Isolated cells were transferred onto poly-D-lysine-coated (200 μg/mL, Sigma Aldrich) coverslips and cultured in TNB 100 medium supplemented with TNB 100 lipid protein complex, 100 μg/mL streptomycin, and penicillin (all from Biochrom, Berlin, Germany) at 37 °C and 5% CO_2_. To avoid neurite outgrowth, which could cause variations in expressed types and amounts of current, and to circumvent space clamp problems, the DRG neurons were studied within 24 h after plating [15,67].

HEK293 cells stably transfected with human Kcnt1 (referred to as HEK-Slack cells; SB-HEK-KCa4.1; SB Drug Discovery, Lanarkshire, UK) were cultured in minimum essential medium with 10% fetal calf serum, supplemented with 2 mM L-glutamine and 0.6 mg/mL G-418 (all from Gibco/Thermo Fisher Scientific) at 37 °C and 5% CO_2_. To obtain HEK-Slack cells that additionally express P2X3 (referred to as HEK-Slack-P2X3 cells), transient transfections of human P2X3 (NM_002559) were done with a pIRES2-EGFP vector plasmid (kindly provided by Prof. Dr. Peter Illes, University of Leipzig, Germany) and Roti-Fect transfection reagent (Carl Roth, Karlsruhe, Germany) according to the manufacturer’s instructions. Cells were split every 3 to 4 days and seeded 2 days before experiments.

Whole-cell voltage clamp recordings were conducted with an EPC 9 amplifier combined with Patchmaster software (HEKA Electronics, Lambrecht/Pfalz, Germany). Currents were filtered at 5 kHz and sampled at 20 kHz. Offline analyses were performed using the Fitmaster software (HEKA Electronics). I_K_ were elicited by protocols consisting of 500 ms-long test pulses ranging from −120 to +120 mV in steps of 20 mV. The holding potential was −70 mV. The pipette solution contained 140 mM KCl, 2 mM MgCl_2_, 5 mM EGTA, and 10 mM HEPES and was adjusted to pH 7.4 with KOH. The physiological external solution contained 140 mM NaCl, 5 mM KCl, 2 mM CaCl_2_, 2 mM MgCl_2_, and 10 mM HEPES and was adjusted to pH 7.4 with NaOH. In a Na^+^-free external solution, NaCl was replaced with 140 mM choline chloride. In a Ca^2+^-free external solution, CaCl_2_ was replaced with 2 mM MgCl_2_ to obtain a final concentration of 4 mM MgCl_2_. Patch pipettes were fabricated with borosilicate glass (Science Products, Hofheim am Taunus, Germany) using a conventional puller (Flaming/Brown Micropipette Puller, Sutter Instruments, Novato, USA) and heat-polished to give a pipette resistance of 3–5 MΩ. Shortly before a coverslip was mounted for recordings, it was dipped in extracellular solution containing 10 μg/mL fluorescein isothiocyanateconjugated IB4 (Sigma-Aldrich) for 5–10 min, and only IB4-binding DRG neurons were analyzed. All recordings were made at 37 °C. α,β-meATP (Sigma-Aldrich) was added with a pipette to the bath chamber to reach a final concentration of 30 µM [29], and I_K_ was recorded 5 min after α,β-meATP administration.

### 4.7. Calcium Imaging

Calcium imaging experiments were performed 24 h after DRG preparation [63]. Neurons were loaded with 5 µM Fura-2-AM-ester (Biotium, Fremont, USA) in supplemented Neurobasal Medium for 45 min at 37 °C, transferred to the perfusion chamber, and continuously superfused with a physiological Ringer solution (145 mM NaCl, 1.25 mM CaCl_2_, 1 mM MgCl_2_, 5 mM KCl, 10 mM Glucose, and 10 mM HEPES, pH 7.4, adjusted with NaOH). For calcium imaging, a Nikon Eclipse Ts2R inverse microscope equipped with a complete illumination system (DG4, Sutter Instruments), a Hamamatsu digital camera (ORCA-05G), Fura-2 filters, and a motorized microscope stage (Märzhäuser Wetzlar, Wetzlar, Germany) was used. Images were taken every 2 s at two wavelengths (340 and 380 nm) and were processed using the NIS-Elements software (Nikon). Baseline measurements were performed in Ringer solution at a flow rate of 1–2 mL/min. For stimulation, 30 μM α,β-meATP dissolved in Ringer solution was applied by bath perfusion for 30 s at room temperature. At the end of each measurement, cells were stimulated for 20 s with 75 mM KCl to identify viable neurons for evaluation. A calcium response was defined as a simultaneous increase at 340 nm and a decrease at 380 nm, when the 340/380 nm ratio normalized to baseline exceeded 20% of the baseline level. Acquired images were displayed as the 340/380 nm ratio.

### 4.8. Experimental Design and Statistical Analyses

Data are expressed as mean ± SEM. All statistical analysis was performed with GraphPad Prism version 8 for windows. *p* values < 0.05 were considered statistically significant. Single comparisons were performed using Student’s t-test. For behavioral experiments, two-way ANOVA (time x genotype) was used to measure effects across time between groups. Multiple comparisons between groups were performed using Sidak’s correction. Numbers of experiments (cells or mice) and statistical results are provided in the results section and the figure legends.

## Data Availability

Data is contained within the article or supplementary material.

## Supplementary Materials

The following are available online at https://www.mdpi.com/1422-0067/22/1/405/s1.

## Abbreviations

α,β-meATP	α,β-Methylene adenosine 5′-triphosphate
AF488	Alexa Fluor 488
CGRP	Calcitonin gene-related peptide
DRG	Dorsal root ganglion
GFP	Green fluorescent protein
IB4	Griffonia simplicifolia isolectin B4
I_K_	K^+^ current
I_KNa_	Na^+^-activated outward K^+^ current
I-V	Current-Voltage
NF200	Neurofilament 200
PBS	Phosphate-buffered saline
qRT-PCR	Quantitative real-time reverse transcription PCR
RIPA	Radioimmunoprecipitation assay
Slack	Sequence like a Ca^2+^-activated K^+^ channel
SNI	Spared nerve injury
TUBB3	Tubulin β 3
WT	Wild-type
