# Supplementary material for: Functional Coupling of Slack Channels and P2X3 Receptors Contributes to Neuropathic Pain Processing
Source: Int J Mol Sci. 2021 Jan 2;22(1):405. doi: 10.3390/ijms22010405 (PMC7795269; doi:10.3390/ijms22010405)
Supplement: Supplementary file 1 [file ijms-22-00405-s001.pdf]

Supplementary informaion

Functional coupling of Slack channels and P2X3 receptors contributes to neuropathic pain processing

Ruirui Lu,[1,*] Katharina Metzner,[1] Fangyuan Zhou,[1] Cathrin Flauaus,[1] Annika Balzulat,[1] Patrick Engel,[1] Jonas Petersen,[1] Rebekka Ehinger,[2] Anne Bausch,[2] Peter Ruth,[2] Robert Lukowski,[2] and Achim Schmidtko[1]

[1] Institut für Pharmakologie und Klinische Pharmazie, Goethe-Universität Frankfurt am Main, 60438 Frankfurt am Main, Hessen, Germany
[2] Pharmakologie, Toxikologie und Klinische Pharmazie, Institut für Pharmazie, Universität Tübingen, 72076 Tübingen, Baden-Württemberg, Germany
* Correspondence: Lu@em.uni-frankfurt.de; Telephone: +49-69-798-29377 (R. Lu).

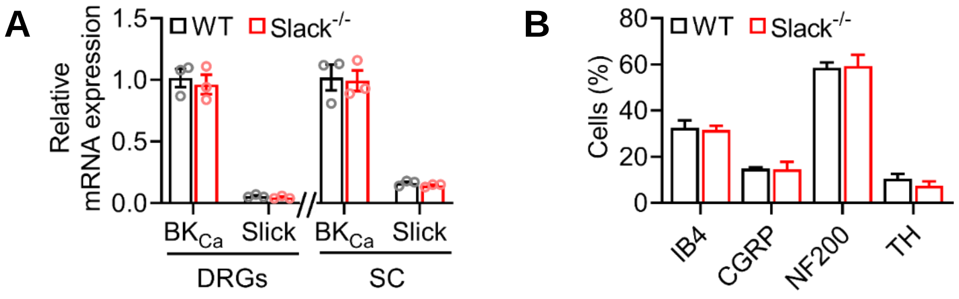

Figure S1.

(A) Expression of BKCa mRNA and Slick mRNA in dorsal root ganglia (DRGs) and the spinal cord (SC) from wild-type (WT) and Slack$^{-/-}$ mice. Multiple t tests; p = 0.6467, 0.3486, 0.8526, and 0.2219, respectively.

(B) Percentages of DRG neurons binding isolectin B4 (IB4) or immunoreactive for calcitonin gene-related peptide (CGRP), neurofilament 200 (NF200) or tyrosine hydroxylase (TH) are similar in WT and Slack$^{-/-}$ mice. Multiple t tests; p = 0.8105, 0.9366, 0.9122, and 0.3266, respectively.

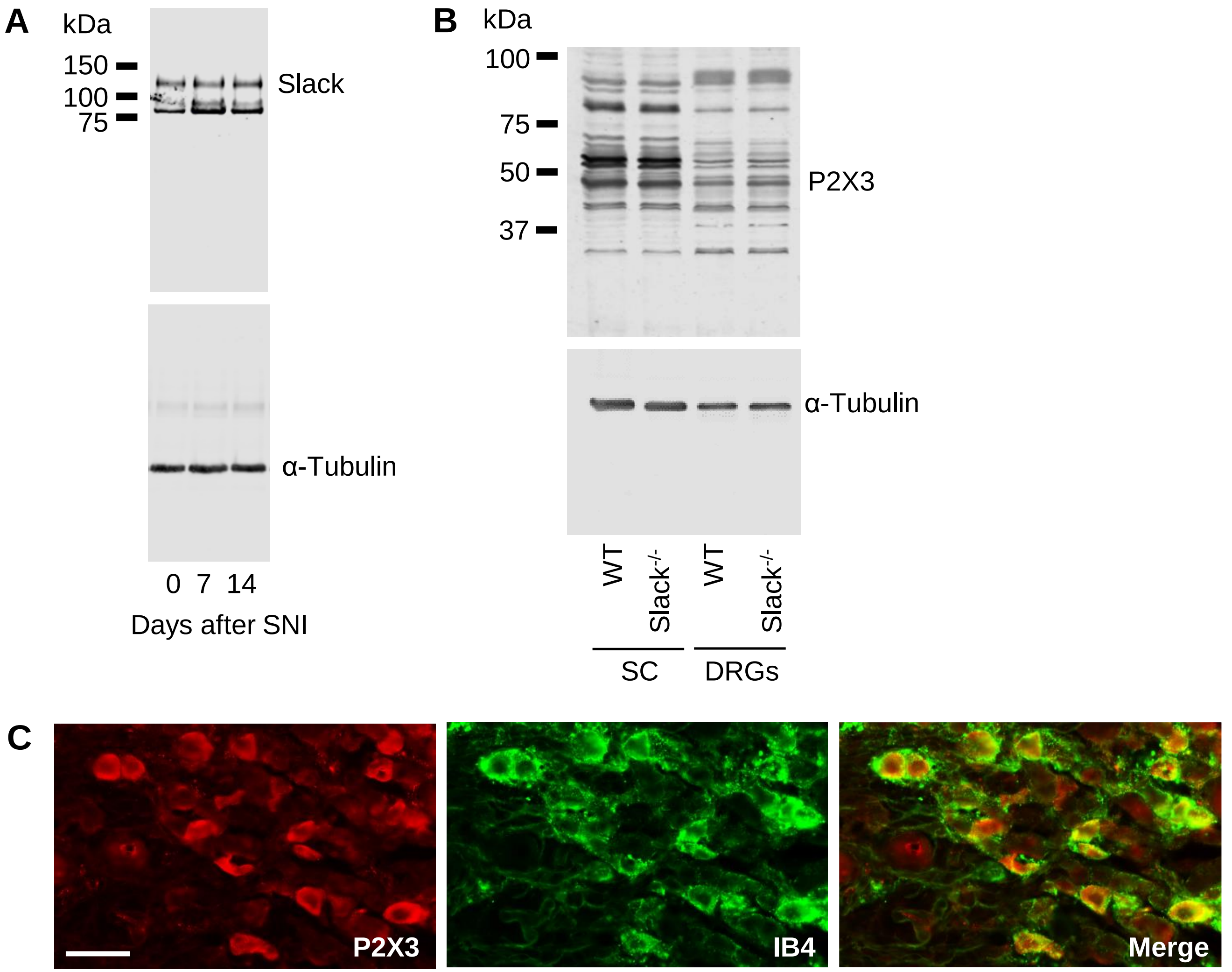

Figure S2.

(A) Western blot of spinal cord extracts show similar Slack protein expression in naive mice (0) and 7 or 14 days after SNI surgery. Uncropped original image of Figure 3E.

(B) Western blot of P2X3 in spinal cord (SC) and DRGs from WT and Slack$^{-/-}$ mice. Uncropped original image of Figure 4E.

(C) Double-labeling immunostaining of P2X3 and binding of IB4 in DRG neurons revealed that the vast majority of P2X3-positive neurons bind IB4. Scale bar: 50 µm.
